# Co-infection of Mandrills with SIVmnd and STLV-1: implications for clinical outcome

**DOI:** 10.1186/1742-4690-11-S1-P41

**Published:** 2014-01-07

**Authors:** Sandrine Souquière, Mirdad Kazanji

**Affiliations:** 1 Laboratoire de Rétrovirologie, Centre International de Recherches Médicales de Franceville (CIRMF), Franceville, Gabon; 2 Institut Pasteur de Bangui, Bangui, Central African Republic

## 

We showed previously that Mandrills infected with STLV have a variable proviral load and evidence of CTL-specific responses. STLV-infected mandrills show an activated immune system with no clear evidence of clinical manifestation or associated illness. Mandrills are also naturally infected with SIVmnd, with no T cell activation or CD4^+^ T cell depletion, despite a high viral load. In humans, dual infection with HIV and HTLV-1 may worsen clinical related outcomes. Our aim was to evaluate the effect of coinfection with SIVmnd and STLV-1 on viral burden, immunological changes and clinical outcome in Mandrills. We showed that SIV viral load was slightly higher in SIV-infected mandrills than in SIV+/STLV+ animals. In contrast, STLV-1 proviral load was higher in co-infected monkey than mono-infected group. Furthermore, the dually infected mandrills have statistically lower CD4+ T cells (*p* < 0.01). Coinfection was also associated with a decrease in the proportion of naive CD8+ T-cells (*p* < 0.001) and an increase in that of central memory cells (*p* < 0.05). A lower percentage of Ki67 was found in CD4^+^ and CD8^+^ T cells from SIV-infected animals than in the other groups. However co-infected monkeys have had the higher percentages of activated CD4^+^ and CD8^+^ T (*p* < 0.01). In the coinfected group, we identified two mandrills with high immune activation and clonal integration of STLV provirus. These animals presented pathological manifestations (infective dermatitis and generalized scabies) rarely encountered in nonhuman primates. These results highlight the importance of multiple infections in nonhuman primates.

